# Complete mitochondrial genome of sea peach *Halocynthia aurantium* (stolidobranchia: Pyuridae) from Korea

**DOI:** 10.1080/23802359.2021.1893618

**Published:** 2021-03-18

**Authors:** Jong-Oh Kim, Seong Seok Choi, Yong Bae Seo, Jiyoung Shin, Ji-Young Yang, Gun-Do Kim

**Affiliations:** a Institute of Marine Life Science, Pukyong National University, Busan, Republic of Korea; b Research Institute for Basic Science, Pukyong National University, Busan, Republic of Korea; c Department of Food Science & Technology, Pukyong National University, Busan, Republic of Korea; d Department of Microbiology, Pukyong National University, Busan, Republic of Korea

**Keywords:** *Halocynthia aurantium*, mitochondrion genome, phylogenetic analysis, Pyuridae

## Abstract

*Halocynthia aurantium* (Stolidobranchia: Pyuridae) is a species of tunicate of commercial value that is commonly found in the northern Pacific Ocean and in the Bering Sea. Here, we determined the complete mitogenome of sea peach *H. aurantium* using 150 PE high-throughput sequencing. The assembled mitogenome is 14,979 bp in length (overall A + T contents 56.2%), and contains 13 protein-coding genes, 21 transfer RNAs, two ribosomal RNAs. Phylogenetic analysis of the mitogenome sequence of *H. aurantium* fully resolved it in a clade with *H. roretzi*. These data and results will be useful for future studies on the evolution of the *Halocynthia* and the Pyuridae.

*Halocynthia aurantium* (Pallas, 1987) is found in the northern Pacific Ocean and in the Bering Sea. This tunicate is usually called the sea peach because of its superficial resemblance in color and shape to a peach. It is an economically valuable marine benthic organism in the East Sea, Korea (Lee et al. 2009). *Halocynthia aurantium* is widely consumed in East Asia and is attracting attention as a potentially new aquaculture variety in Korea. Although some studies on the physiological characteristics required for farming conditions have been conducted (Baik et al. 1986;Honegger and Koyanagi 2008; Lee et al., 2009), research on its genetic structure are lacking. The aim of this study is to document and characterize the mitochondrial genome of *H. aurantium* and to provide an evolutionary context within the Pyuridae.

The voucher specimen (Sample no. MFDS-SMI02) was caught in the East Sea in Korea (38°18′58.2″N 128°33′19.9″E) and was deposited at the Department of Food Engineering, Pukyong National University (Ji-young Yang, jyyang@pknu.ac.kr). The DNA was extracted using DNeasy Blood and Tissue Kit following the manufacturer instruction (Qiagen, Germany). The DNA library was constructed with the MGIEasy DNA Library Prep Kit (MGI, China) and sequenced using 150 bp paired-end reads by MGIseq system (MGI). The 30,741,119 reads were cleaned by Cutadapt ver. 1.9 (Martin 2011) before *de novo* assembly using CLC Genomics Workbench (Qiagen, Germany). The annotation was performed using the MITOS Web Server (Bernt et al. 2013). The phylogenetic relationship within the Pyuridae was analyzed with MEGA-X ver. 10.1.5 (Kumar et al. 2018) using the maximum-likelihood criterion and Tamura-Nei model with 1000 bootstrap replicates.

The complete mitogenome of *H. aurantium* is 14,979 nucleotides in length and contains 13 protein-coding genes (PCGs), two ribosomal RNA (rRNAs), 21 transfer RNA (tRNAs), which are all encoded on the H-strand. Among the 13 PCGs, ND3 was initiated with the incomplete start codon (TTA) and ND4L has an incomplete stop codon (T). The overall base composition of the mitogenome is A 22.8%, T 42.4%, G 24.4% and C 10.5%, with a high A + T content of 65.2%. The assembled sequence and detailed information of the mitogenome was submitted to GenBank (Accession No. MT827076). From the constructed phylogenetic tree using published six mitogenome sequences in the Pyuridae, *H. aurantium* was fully resolved in a clade with *H. roretzi* (Accession No. NC002177), sister to *H. papillosa* (Accession No. FM177863) (Figure 1). These phylogenetic results support previous report based on 18S rDNA and COI sequences of Pyuridae (Pérez-Portela et al. 2009).

**Figure 1.**
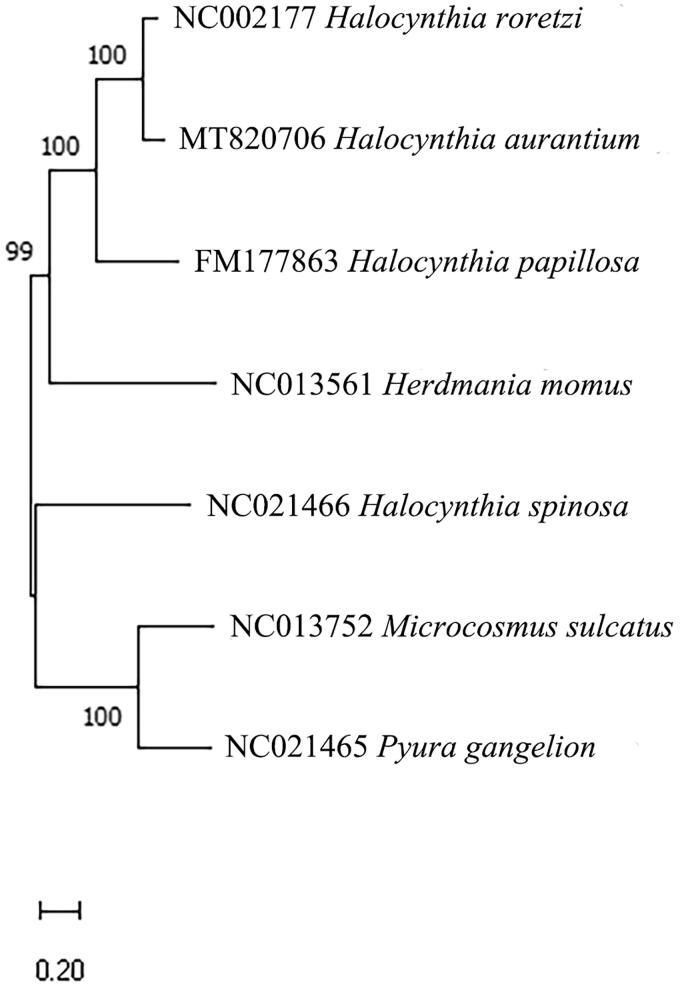
Phylogenetic tree based on *Halocynthia aurantium* and six related mitogenome sequences classified in the Pyuridae. The 6 mitogenome sequences from GenBank and the *H. aurantium* mitogenome sequence were aligned using ClustalW and the phylogenetic analysis was conducted using the Maximum Likelihood optimality criterion with 1000 bootstrap replicates. The percentage at each node represents the bootstrap values.

## Data Availability

The genome sequence data that support the findings of this study are openly available in GenBank of NCBI at (https://www.ncbi.nlm.nih.gov) under the accession no. MT827076. The associated BioProject, SRA, and BioSample numbers are PRJNA701138, SRP305601, and SAMN17849029, respectively.
